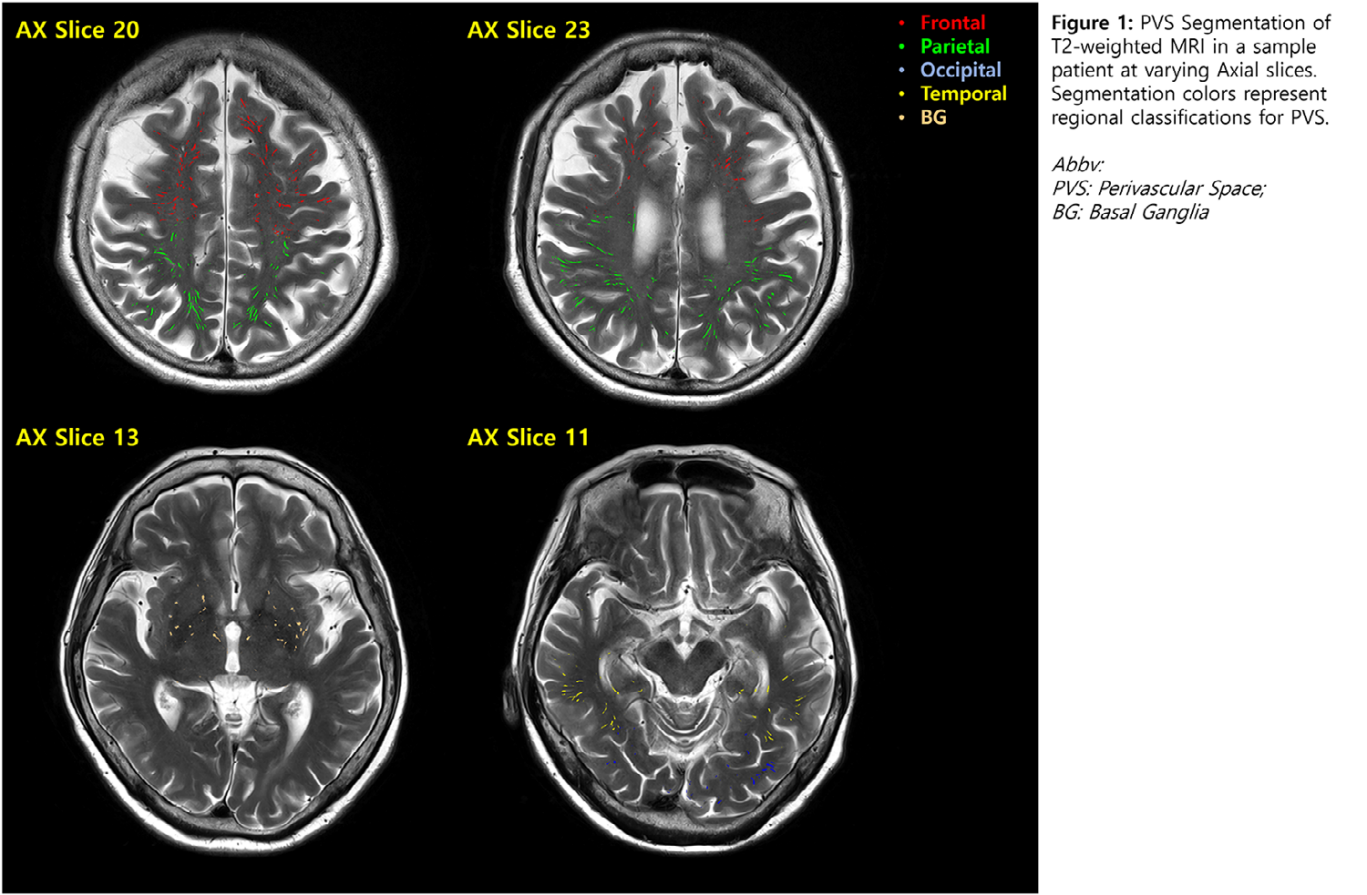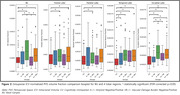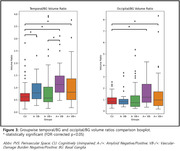# Distinct Association of Amyloid and Vascular Pathologies with Enhanced Perivascular Spaces

**DOI:** 10.1002/alz.087217

**Published:** 2025-01-09

**Authors:** Woo Sik Kim, Roh‐Eul Yoo, Yejin Hwang, Yelim Yang, Joon‐Kyung Seong, Seung Hong Choi, Wha Jin Lee

**Affiliations:** ^1^ NeuroXT, Seoul Korea, Republic of (South); ^2^ Seoul National University College of Medicine, Seoul Korea, Republic of (South); ^3^ Seoul National University Hospital, Seoul Korea, Republic of (South); ^4^ Korea University, Seoul Korea, Republic of (South)

## Abstract

**Background:**

Glymphatic system dysfunction as characterized by increased MRI‐visible Perivascular Spaces (PVS) is speculated to play a role in the acceleration of amyloid accumulation in Alzheimer’s Disease (AD). However, while PVS is also prevalent amongst Vascular Dementia (VD), the pathological distinctions between regional PVS in AD‐ and VD‐driven cohorts remain largely unknown. Through a mixed dementia cohort, we examined these pathology‐driven localization patterns via automated PVS segmentations from T2‐weighted MRI.

**Method:**

99 cognitively unimpaired (CU) and 190 cognitively impaired (CI) patients’ data were collected from the Seoul National University Dementia cohort. Through visual assessments of individual’s ^18^F‐Florbetaben PET, FLAIR and SWI images, expert radiologists classified CI patients into 4 groups based on both amyloid and vascular‐damage burden (26 A‐VB‐, 63 A‐VB+, 26 A+VB‐, and 75 A+VB+). PVS segmentation involved masking and thresholding hyperintense vessel structures using slice‐wise Frangi filters applied to T2‐weighted MRIs at the basal ganglia (BG) and cerebral white matter (WM), segmented into 4 major lobes (frontal, parietal, temporal, and occipital). We calculated PVS volume fractions (PVS‐VF) as voxel counts normalized by intracranial volumes. Automated segmentations were validated against manually segmented PVS volumes at BG and whole WM (rank correlation coefficients: 0.634, 0.539). Groupwise differences were calculated through two‐sample t‐tests corrected for multiple comparisons.

**Result:**

For A+ groups, significant increases in PVS‐VF were observed at the temporal and occipital lobes as compared to CU, a finding that agree well with prior visual rating studies. However, VB+ groups showed significant PVS‐VF increases across all lobes as compared to CU. Particularly, BG represented the most pronounced VD‐related PVS, with a significantly greater PVS‐VF in the VB+ groups compared to their VB‐ counterparts. Interestingly, PVS volume ratios of lobar regions and BG showed no significant increases for A‐VB+, while all A+ groups showed significant increases in temporal and occipital regions as compared to CU.

**Conclusion:**

Full utilization of PVS as a core biomarker in discerning glymphatic dysfunctions of AD requires full understanding of their properties in those contexts. Cross‐sectional differentiation of regional PVS localization in AD can become a steppingstone into elucidating those associations within the AD continuum.